# *Rhythms of the Earth*—Editorial Introduction

**DOI:** 10.1029/2023GH000815

**Published:** 2023-04-10

**Authors:** Karim‐Aly S. Kassam, Morgan Ruelle, Christopher P. Dunn, Raj Pandya, Felice Wyndham

**Affiliations:** ^1^ Department of Natural Resources and the Environment American Indian and Indigenous Studies Program Cornell University Ithaca NY USA; ^2^ International Development, Community & Environment Clark University Worcester MA USA; ^3^ Cornell Botanic Gardens Cornell University Ithaca NY USA; ^4^ Thriving Earth Exchange American Geophysical Union Washington DC USA; ^5^ School of Anthropology & Museum Ethnography University of Oxford Oxford England

## Abstract

This special issue of *GeoHealth*, entitled *Rhythms of the Earth: Ecological Calendars and Anticipating the Anthropogenic Climate Crisis*, is a transdisciplinary articulation of a methodology of hope to confront the multiple injustices of the Anthropocene. One of the greatest challenges of the climate crisis is the lack of predictability at the scale of communities where impacts are most immediate. Indigenous and rural societies face an ever shifting “new normal” through increasing inconsistency in the seasonality of temperature and precipitation, as well as greater frequency of extreme weather events. With global food systems dependent on local and small producers, climatic variability disrupts access to affordable, nutritious, and culturally relevant food. Ecological calendars are context‐specific knowledge systems grounded in a particular cultural milieu and ecological space, that build anticipatory capacity for seasonal change. They measure and give meaning to time. Based on close observation of one's habitat, human societies have used such calendars for hundreds of years and potentially millennia. By engaging with the interactions among physical phenomena (such as the first snowfall or last frost) and biological events (such as blossoming of specific trees, arrival of migratory birds or mammals, appearance of plants or insects), human societies have been able to identify optimal time windows for their livelihood activities. The 11 research articles in *Rhythms of the Earth* cover a considerable geographical breadth from Africa to the Arctic; and, from North and South America to Central Asia. They provide evidence that spans millennia from the Roman Empire to the contemporary Anthropocene.

## Key Points

- *Rhythms of the Earth* is a transdisciplinary collaboration between Indigenous Knowledge and Science
- In *Rhythms of the Earth* , an innovative culturally and ecologically grounded way to anticipate seasonal change is proposed
- Significant diversity of thought from around the world is engaged in *Rhythms of the Earth* because the research articles are co‐authored by artists, Indigenous community members, and scholars in the biological, physical, and social sciences as well as the humanities

## 1 Multiple Injustices

The climate crisis forces humanity to fundamentally examine and respond to the historic and contemporary prevalence of injustice. Industrial human societies are not just a sociocultural and an ecological force, but also a geological force; hence, the name of our age: the Anthropocene. The Anthropocene, the age of humankind, is uneven in its impact. While the term suggests blanket human responsibility, the causes of climate change have been perpetrated by specific industrial ideologies espoused by the command and market economies of a small group of countries and implemented by identifiable human societies. The recent COP 27 Final Agreement, establishes “loss and damage” and “adaptation” funds for over 100 nations that contributed little or nothing to the cumulative carbon emissions in the atmosphere; but these are merely a first step in public realization and acknowledgment of culpability (UNFCCC, 2022). The injustices of climate change are taking place across several interacting scales. We, academics and our students, are therefore compelled to examine and thoughtfully respond to the complex injustices that the climate crisis vividly exposes.

First, the “slow violence” of the climate crisis exacerbates existing inequities (Nixon, 2013; Rice, 2016). The Anthropocene is a legacy of colonization driven by industrializing cultures with dire outcomes for “other” people, their habitats, and even their minds. Its impacts are greatest on Indigenous and rural societies as well as vulnerable groups within urban communities who remain willfully invisible to mainstream societal structures and their attendant communications media. Yet, these communities have historically and currently contributed the least to climate change. Paradoxically, Indigenous and rural societies are at the vanguard in terms of solutions to climate problems because they have retained ecological relations within, and their cultural survival depends upon, their respective environments. These communities continue to confront the trauma of colonization and cultural genocide while dealing with the cumulative burden of climate change and its emergent consequences.

The second layer of injustice is that the climate crisis is arguably a crime perpetrated against the planet. It furthers the “death of birth” (Wilson, 1999) in which extinction is outpacing evolution (IPBES, 2019). Significant and large‐scale environmental degradation has led to a massive loss of biodiversity that jeopardizes all life's potential and ability, including human ingenuity to withstand environmental stress. In fact, biological extinctions and erosion of biological diversity, driven largely by the consumption mentality that produced the climate crisis, are also leading to loss of cultural and language diversity, key elements of our humanity (Dunn, 2018). Consequently, the diverse forms and ways of being that facilitate life on the planet are being eliminated, thereby limiting future possibilities and opportunities to adapt and evolve.

Finally, the third element of injustice, linked to the second, is the undermining of future generations of humans whom we have yet to meet and know. Specifically, future generations of people who could learn how to live in close relation to the land and potentially repair the harm perpetrated by their ancestors.

The climate crisis places significant responsibility on contemporary scholars in the humanities as well as biological, social, and physical sciences. Yet, our ethics remain consciously flawed as our “exosomatic instruments” (Georgescu‐Roegen, 1966, 1971), the technological tools that extend our bodily reach across physical space and historical time, outpace our reflective capabilities of consequences. It is no longer sufficient for scholars to point out what is unashamedly unjust. Most academics and research scientists, irrespective of their disciplinary or ethnic backgrounds, have benefitted and continue to profit from industrial infrastructure. In order to advance our careers, we—without acknowledging cognitive dissonance—embrace technologies in classrooms, laboratories, and libraries, not to mention air travel for research and to share findings at conferences, that run on the infrastructure developed from the industrial revolution and are primary drivers of the Anthropocene.

Therefore, we are compelled to engage these inconvenient realities with the necessary intellectual and ethical rigor, not only for the well‐being of our students and communities with whom we work, but for all life, to which we are fundamentally connected. In very tangible ways, this life facilitates our food systems, and less tangible but equally important ways, it is a source of peace and mental health in the sublime web of connectivity of our habitat.

### 1.1 A Methodology of Hope: Ecological Calendars

This special issue of *GeoHealth*, entitled *Rhythms of the Earth: Ecological Calendars and Anticipating the Anthropogenic Climate Crisis*, is a transdisciplinary articulation of a methodology of hope to confront the multiple injustices of the Anthropocene.

One of the greatest challenges of the climate crisis is the lack of predictability at local scales where impacts are most immediate. Indigenous and rural societies face an ever shifting “new normal” through increasing inconsistency in the seasonality of temperature and precipitation, as well as greater frequency of extreme weather events. Meanwhile, our measurements are inadequate to capture what is already happening, let alone help people plan for what comes next. At the local level, the impacts of anthropogenic climate change are not felt as “averages” in temperature. Nor is precipitation homogenous—it has character and nuance that holds specific meaning and has an impact on people's livelihoods and their habitat. To put it succinctly, these are concrete events with real consequences. With global food systems dependent on local and small producers (Lowder et al., 2021), climatic variability disrupts access to affordable, nutritious, and culturally relevant food.

Ecological calendars are context‐specific knowledge systems grounded in a particular cultural milieu and ecological space, that build anticipatory capacity for seasonal change. They present a biophysically and culturally relevant alternative to anticipating climate change. Ecological calendars are a manifestation of embodied knowledge systems that measure and give meaning to time based on close observation of one's habitat (Kassam et al., 2018, p. 250, 2021, pp. 510–511). Human societies have used such calendars (often in combination with solar or lunar calendars) for hundreds of years and potentially millennia (Kassam et al., 2011; Tally‐Schumacher, 2023). By engaging with the interactions among physical phenomena (such as the first snowfall or last frost) and biological events (such as blossoming of specific trees, arrival of migratory birds or mammals, appearance of plants or insects), human societies have been able to identify optimal time windows for their livelihood activities. Compared to the mechanistic and rigid solar‐based Gregorian and Julian calendars, these organic and relational articulations of biophysical occurrences reveal patterns among seasonal events that provide flexibility of response times. Our aim is not to diminish the value of solar or lunar calendars; rather, to show that understanding relations among these biophysical phenomena has the potential to simultaneously build mitigative and adaptive capacity to climate change by generating anticipatory capability (Ullmann & Kassam, 2022).

*Rhythms of the Earth* brings together various disciplinary insights within the biological, social, and physical sciences as well as the humanities. However, it is not sufficient that students and academics engage in applied research without recognizing the limitations of their epistemological foundations and their own expertise. We must, with humility, accept the agency, experiences, and knowledge of the Indigenous and rural societies with whom we work. Foundational to ecological calendar research as a methodology of hope to the climate crisis, is the inclusion of multiple ways of knowing. By drawing on the diverse ways of being through the ecological knowledge of farmers, fishers, herders, hunters, orchardists, and the like, we seek to cocreate ecological calendars that are effective locally and applicable in the context of the climate crisis.

## 2 Impetus for the *Rhythms of the Earth*

The impetus for this work began with human ecological research among the Iñupiat and Inuvialuit in the Western Arctic of North America in the late 1990s. It was clear then that changes to sea‐ice were going to drastically affect the food system of marine mammals, including humans who depend on it for their nutritive needs. Hunting, fishing, and gathering are not relics of a bygone era, they are realities in the twenty‐first century and key to food security for modern and dynamic Indigenous populations in the circumpolar Arctic. These practices are not just testimony to cultural continuance but the basis of survival. The impacts of the climate crisis on the food system made it clear that there was a need to address this issue by building anticipatory capacity to seasonal change (Kassam, 2009a; Kassam et al., 2018).

In 2006, what had already become apparent in the Arctic, was reiterated in our research in the Pamir Mountains of Central Asia (Kassam, 2009b). In these areas, the Indigenous populations are primarily engaged in farming, herding, gathering of medicinal plants, and to a lesser degree hunting and fishing to meet their nutritional needs. The impacts of the climate crisis were already obvious with respect to the food system in this region.

The anxiety we encountered in these distinct Indigenous Alpine and Arctic regions was palpable, providing momentum for collaborative transdisciplinary applied research to build anticipatory capacity. Much of climate research is instrumental and detached from local context, using satellites, weather data, and models to assess climate change. We conducted in‐situ, applied research where physical, biological, and social scientists engage Indigenous peoples in knowledge co‐creation in their homelands. Under these circumstances, the anxiety felt by Indigenous peoples is shared with a community of researchers. In such a reality, the impacts and injustices of climate change are tangible, immediate, and deeply disturbing to academics and their students alike. As a result of this collaborative and co‐generative process of working together with Indigenous peoples, scholars will then communicate this anxiety and its implications to a wider audience of scholars and the public.

In 2006, in the Bartang Valley of the Gorno Badakhshan Region of the Tajik Republic we learned about specific ecological calendars, which community members referred to as the “calendar of the human body” (Kassam, 2009a; Kassam et al., 2011). The very idea of a calendar measuring time using the human body suggests embodied knowledge where villagers' performative livelihood actions reflect cumulative intergenerational knowledge. These calendars have been used for centuries by diverse ethnic groups in varied ecological zones addressing different ecological professions to anticipate seasonal change. Under Soviet rule, use of these calendars was suppressed, hence the knowledge related to these calendars fell into disuse. Between 2006 and 2009, we tried to learn more about these calendars through a review of ethnographic documents and ancient manuscripts. From 2010 to 2013, we collaborated with physical and biological scientists who were willing to undertake in‐situ participatory applied research to build anticipatory capacity. In addition to specific disciplinary expertise, we sought out scientists and students who had an ethical quality that is fundamental to effective research: the ability and humility to work with Indigenous and local communities and valorize their context‐specific ecological knowledge.

In 2014, following a recommendation from Cliff Duke, the Director of Science Programs at the Ecological Society of America, the Thriving Earth Exchange of the American Geophysical Union (AGU) approached our research group at Cornell University. Raj Pandya, Vice‐President of Community Science at the AGU called and said: “We hear that you need assistance with climate research among Indigenous communities; tell us what your community partners and you need?” This set into motion a transdisciplinary effort driven significantly by the needs and priorities of our community partners with respect to livelihood and food security related to the climate crisis.

We began by identifying the dimensions of research and collaboration needed to revitalize ecological calendars that had fallen into disuse due to colonialism and cultural genocide. We collaborated with the Massachusetts Institute of Technology's Climate Co‐Lab to crowdsource insights among interested scholars from the biological, physical, and social sciences as well as the humanities. More importantly, this group of researchers needed to be open to meaningfully and respectfully engage with the depth of Indigenous knowledge present among Indigenous communities. We also needed to articulate an action research methodology that would allow us to contribute approaches and insights from our diverse fields to cogenerate ecological calendars with our community partners.

First, we chose to establish a proof‐of‐concept in order to access the international collaboration and funding needed. In 2015, we sought support from Cornell University's Atkinson Center for Sustainability's Academic Venture Fund (AVF) which is specifically designed for cutting‐edge transdisciplinary engagements. Upon successfully articulating this proof‐of‐concept and receiving support for research in the United States, we sought international funding from the Belmont Forum to undertake research on ecological calendars. In 2016, we secured funding for Ecological Calendars for Climate Adaptation Project (ECCAP). While the stimulus for this Special Issue entitled *Rhythms of the Earth* began over two decades ago, this specific research on ecological calendars was started in 2015.

## 3 The Insights That Link the Contributing Articles

This Special Issue includes findings from diverse geographical contexts in Indigenous and rural North America and Central Asia. However, we have not limited *Rhythms of the Earth* to the work of the AVF and ECCAP projects. Instead, we have sought to enrich our understanding of ecological calendars by inviting colleagues to share the fruits of their collaborative work with communities in different parts of the globe. We invited Indigenous community members, artists, students, social and biophysical scientists, and scholars from the humanities to jointly author and contribute their insights to the transdisciplinary work on ecological calendars. Thus, the Special Issue demonstrates two important attributes of ecological calendars: first, their universality with respect to diverse human societies across time and space; and second, their relevance within a particular cultural, ecological, and geographic context to anticipate change.

When words fail us in conveying the depth of injustice experienced, humanity often turns to art for its communicative and transformative potential. We begin the discussion of ecological calendars through artistic expression. In October 2021, we organized the *Rhythms of the Land* International Conference, which brought together more than 80 participants, including Indigenous community members, policy makers, scholars of various disciplines, and students. Works by 20 artists from around the world were exhibited at the Johnson Museum of Art at Cornell University. In “Art and Environmental Struggle Curating an Exhibition About Place‐Rooted Ecological Knowledge,” a group of museum curators along with visual and performance artists explore the impact of the climate crisis (Avril et al., 2022). In conversation with the exhibit described above, in “Interpreting Ecological Calendars for the Public Through Exhibits, Art, and Education” a group consisting of horticulturists, public educators, graphic designers, artists, and students at the Cornell Botanic Gardens turned our ecological calendars research into visual art for interpretation and translation to engage the public (Skelly et al., 2022). In fact, Botanic gardens are increasingly focusing on expressions of biocultural diversity conservation, such as ecological calendars, to activate the visiting public (Dunn, 2012, 2017). This exhibit also coincided with the International Conference. The objectives of these two art exhibits were to connect “struggle” with “hope” and find ways to engage the public in reflecting about their habitat in more immediate and intimate ways within their daily lives. Art, rather than academic literature, was and continues to be the most effective way to begin a conversation with diverse peoples and their distinct experiences and perspectives.

*Rhythms of the Earth* contains articles related to ecological calendars from the Arctic to North Africa, ranging in time from the Roman Empire to contemporary Central Asia. The Special Issue includes archeological evidence of ecological calendars in regions of the Roman Empire, specifically the Mediterranean approximately 2000 years ago (Tally‐Schumacher, 2023). In “Warm Soil, Westerly Wind, and Wet Feet: Feeling and Measuring Ecological Time in the Roman World,” we are shown historical evidence of the universality of context‐specific insights related to human experience of the seasons across time and space. Furthermore, the widespread use of our modern 365 days and 12‐month calendar actually has its roots in Roman colonization and imperialism. The Romans, like Indigenous peoples around the globe, integrated use of celestial calendars with ecological calendars. Much like Indigenous ecological calendars elsewhere, the Roman calendars incorporated corporeal sensations. For example, warmth experienced from the soil is a common indicator in the calendar of the human body among diverse Central Asian communities historically as well as at present.

The evolution of ecological calendars and the entangled nature of human and ecological systems are illustrated through the history of olive cultivation in Sicily. Drawing on written historical sources, oral histories, and contemporary interviews, Ferrara and Ingemark (2023) compile and share a monthly ecological calendar of the olive tree on the island of Sicily over the last 2,800 years. In “The entangled phenology of the olive tree: A compiled ecological calendar of *Olea europaea* L. over the last three millennia with Sicily” as a case study they offer a compelling example of the interplay between plant behavior and human adaptation by exploring spatial and temporal variations in the timing of key cultivation activities like pruning, grafting, harvesting, and transplanting. They show the long‐term phenological stability of the olive tree in this region and the ways in which the steady accumulation and activation of cultural knowledge through ecological calendars has contributed to that stability. Humans first adapted to plant phenology by scheduling practices in response to the plant's perceived agency—for instance, transplanting in the fall when the plant is focusing its energy on its root system. Human practices then transform plant physiology and behavior, for example, though grafting, genetic selection, and patterns of cultivation that are responsive to physical characteristics like slope, soil, and prevailing winds. The concept of relatedness, then, is a process in which both plants and people can change together. The historic evolution of the ecological calendar of the olive tree provides hope by showing that past ecological and cultural disturbances have turned out to be a strength, as locals have used them to better understand connections between environmental changes and sustainable stewardship practices.

In our contemporary context of the Anthropocene, whose uneven impacts relate directly to colonialism, the imperial Roman legacy simultaneously reveals colonial disempowerment as well as the role of Indigenous or local knowledge to achieve food and livelihood security. This historical and contemporary entanglement cannot be denied nor rendered into simplistic dichotomies which erase nuance and complexity. In this light, the recent speech by President Biden at the Tribal Nations Summit on the significance of Indigenous Knowledge to guide US federal “agency decision making” is simultaneously an acknowledgment by the most powerful nation state, like Imperial Rome, of the weaknesses of its epistemological and ontological frameworks for policy formulation; a potential threat to the continuing co‐option of Indigenous ways of knowing; and a significant recognition that Indigenous people and their stewardship practices matter to human survival in the third millennium (Biden, 2022).

As a methodology of hope, we then focus on the anticipatory potential of ecological calendars based on more contemporary research with Indigenous communities. In “‘When the Wild Roses Bloom’: Indigenous Knowledge and Environmental Change in Northwestern North America” (Turner & Reid, 2022), a senior ethnobotanist with a lifetime of substantive community‐based experience and a younger Indigenous critical thinker provide historical and contemporary evidence of the use of phenological indicators by diverse Indigenous peoples. Knowledge of this rich biodiversity and its stewardship set the stage for a discussion of ecological calendars in our own research work in the Pamir Mountains of Central Asia and the Standing Rock Sioux Nation in North America. Roshorv and Savnob are two villages in the Bartang Valley of Tajikistan where historical use and linguistic evidence of ecological calendars are extant. In “Ecological Calendars of the Pamir Mountains: Illustrating the Importance of Context‐Specificity for Food Security,” a team of young environmental and climate scientists and an Indigenous ethnolinguist (Ullmann et al., 2022) compare the significance of differing ecological indicators in anticipating seasonal change due to the microclimates of these two closely located Pamiri villages. They visually articulate the use of contemporary ecological calendars that contain oral tradition of Indigenous ways of knowing with contemporary climate science. Similarly, an ethnobotanist, whose career emerged from work with the Standing Rock Sioux Nation, in collaboration with his student and director of a tribal health agency (Ruelle et al., 2022) describe how the cocreation of community‐specific ecological calendars reveal diverse knowledge related to traditional foodways. In “Ecological Calendars, Food Sovereignty, and Climate Adaptation in Standing Rock” they describe the implications of cultural genocide to Indigenous ways of knowing and being and how revitalization is both possible and necessary to secure food sovereignty by building anticipatory capacity.

Similarly, in the southern regions of South America, further examples of well‐developed ecological calendars are still in use and have been adjusted over time in response to environmental, socio‐economic, and political forces. Rozzi et al. (2023) provide a detailed historical account of “Biocultural Calendars Across Four Ethnolinguistic Communities in Southwestern South America” with a particular emphasis on artisanal fisher and pastoralist communities. The authors review the destructive nature of colonialism in Chile that resulted in the fragmentation of communities, language, and cultural identity and integrity. What is evident, however, is not just the resilience of their habitat in the face of climate change, but the simultaneous resilience of communities. Because ecological calendars relate to biophysical and cultural contexts, and the synchronicity between rhythms of ecological systems and human livelihoods and activities, the authors refer instead to “biocultural calendars.”

The Indigenous communities featured in our Special Issue are responding to both long‐term trends and increasing variability in the climate. In “Climate Change Impacts Can Be Differentially Perceived Across Time Scales: A Study Among the Tuareg of the Algerian Sahara,” an international team led by an Algerian scholar (Miara et al., 2022) apply a protocol developed by the Local Indicators of Climate Change Impacts initiative to evaluate local knowledge of climate changes in relation to livelihood activities and cultural practices. The authors find that Tuareg participants are more keenly aware of interannual (cyclical) variability, which they attribute to climate change, than they are of longer‐term trends, despite providing evidence of dramatic changes over the past several decades. Nevertheless, these communities are already responding to “new normal” conditions, for example, by planting wheat nearly 2 months later to accommodate a much‐delayed rainy season.

Exercise of food sovereignty to achieve food and livelihood security are central and underlying themes tying together all the contributions in *Rhythms of the Earth* Special Issue. In “Shifting Seasons and Threats to Harvest, Culture, and Self‐identity: A Personal Narrative on the Consequences of Changing Climate,” Arlyn Charlie, a Teetł’it Gwich'in artist, explores how the slow violence of climate change is making traditional ecological calendars unreliable because of changing patterns in animal behavior and the Arctic habitat. These changes contribute to an increased individual risk in hunting and fishing activities central to the local food system which ultimately affects Gwich'in language and culture. The article, co‐authored with scientists, is written in two fonts to differentiate the narrative element from technical explanations (Charlie et al., 2022).

One potential weakness of contemporary ecological calendars is related to the impact of climate change on indicator species, meaning those organisms whose development and behavior are used to measure the passage of time. Biodiversity, which forms the foundation of ecological calendars, is itself threatened. It is possible to imagine a scenario where climatic change is so rapid, that a particular indicator species becomes affected within a span of a season and can no longer serve as a phenological sign. In the “Role of Biodiversity in Ecological Calendars and Its Implications for Food Sovereignty,” the lead guest editor and principal investigator of the AVF and ECCAP projects along with an evolutionary biologist, provide a proof‐of‐concept for an empirical assessment of the resilience of indicator species to anthropogenic climate change (Kassam & Bernardo, 2022). Using three case studies from the Western Arctic (described earlier) as well as ecological calendars from Sary Mogul in the Alai Valley of Kyrgyzstan and Oneida Lake in Upstate New York, USA, they demonstrate how food sovereignty can be mutually informed by Indigenous knowledge and contemporary science. By bringing multiple ways of knowing together, the authors are building greater vigilance capacity to climatic change that is grounded in a variety of specific cultural and ecological contexts, as well as utilizing the diverse disciplinary expertise of institutionalized science. The article demonstrates how institutionalized science, through an informed community of researchers, can work in the service of Indigenous or local communities who implement these insights through practice, thus interrupting the colonial legacy and beginning repair in the Anthropocene.

It is important to keep in mind that archeological (Tally‐Schumacher, 2023), ethnographic (Kassam et al., 2011), and biocultural evidence (Ferrara & Ingemark, 2023; Kassam et al., 2022; Rozzi et al., 2023) shows that ecological calendars have been in use for hundreds of years if not millennia by human societies. Clearly, there has been climatic and environmental change throughout this period. However, Indigenous societies have been able to adapt their calendars and identify different indicator species in tandem with changing rhythms of the earth because their cultures were grounded in their specific ecological context. Similarly, with multiple ways of knowing involving deep Indigenous knowledge in collaboration with institutionalized science, we propose that ecological calendars remain relevant and become even more significant in the Anthropocene.

## 4 Final Thoughts

The choice of *GeoHealth* as the journal for the articles co‐authored by artists, Indigenous community members, students, and scientists is deliberate. In many cases, contributors are both scholars and community members, thereby bringing together multiple ways of knowing within a single individual. Our aim is to extend our conversation to colleagues in the physical sciences as represented by this journal of the American Geophysical Union and whose membership and reach is international. Furthermore, this journal provides open access to Indigenous and rural communities as well as scholars around the world. We invite you—whether you are a scientist, a member of an Indigenous community, or both—to engage with *Rhythms of the Earth* and to critique and improve upon this body of work. Ecological calendars are meant to be both a continuation and celebration of a historical and rigorous approach to anticipate seasonal change. The goal of this Special Issue is to advance a methodology of hope that can help us all adapt to climate change, repair past harm, and prevent future injustice. For this opportunity, we are deeply grateful to the American Geophysical Union and specifically the editorial team at *GeoHealth*.

## Conflict of Interest

The authors declare no conflicts of interest relevant to this study.

## Data Availability

Data were not used, nor created for this research.
